# The 10-year outcomes of the ASR XL Acetabular System: a single-center experience from China

**DOI:** 10.1186/s13018-019-1173-2

**Published:** 2019-05-24

**Authors:** Guojun Jin, Jisheng Ran, Weiping Chen, Yan Xiong, Jiapeng Bao, Lidong Wu

**Affiliations:** 1 Department of Orthopedics, Taizhou First People’s Hospital, 218th Hengjie Road, Taizhou, 318020 China; 2 Department of Orthopedic Surgery, The Second Affiliated Hospital, Zhejiang University School of Medicine, 88th Jie Fang Road, Hangzhou, 310009 China

**Keywords:** Metal-on-metal, Total hip arthroplasty, Metal ion, Survivorship analysis

## Abstract

**Background:**

The revision rate of articular surface replacement (ASR) implants continues to rise in China because of metal debris. However, there are few reports on the clinical results of ASR implants with prolonged follow-up time in China. This study investigated the clinical outcomes and the risk factors of revision surgery in patients with ASR implants.

**Methods:**

In total, 74 patients (74 hips) who underwent primary total hip arthroplasty (THA) with ASR implants over the past 4 to 10 years were retrospectively analyzed. Relevant clinical, radiographic, and biochemical data were examined.

**Results:**

The average follow-up time was 88.46 (range 23–114) months, and the ASR implants of 18 hips (24.3%) were revised. Patients who received revision surgery had worse joint function with significantly lower Harris Hip Score and Western Ontario and McMaster Universities index than non-revision patients (61.11 ± 6.68 vs 85.30 ± 9.16, *p* < 0.001; 61.00 ± 3.83 vs 79.04 ± 14.49, *p* < 0.001; respectively). Higher acetabular abduction angle and serum Co and Cr concentration were significantly relevant to worse joint function as measured by HSS (*p* = 0.018, 0.009, 0.043, respectively). ROC curve analysis was applied to categorize the optimal cutoff values of acetabular abduction angle and serum Cr and Co concentration for revision surgery, which were settled as 47.80°, 98.44 μg/L, and 6.95 μg/L, respectively. Overall survival of the prostheses with high acetabular abduction angle (> 47.80°, HR = 70.145, 95% CI 1.558–3158.213, *p* = 0.029), high serum Cr concentration (98.44 μg/L, HR = 58.956, 95% CI 1.294–2685.203, *p* = 0.036), and high serum Co concentration (> 6.95 μg/L, HR = 179.511, 95% CI 2.360–13656.941, *p* = 0.019) decreased significantly than the lower groups.

**Conclusions:**

Evaluation of the DePuy ASR XL articulation demonstrated increased rates of revision following a longer follow-up period. High acetabular abduction angle and serum Cr and Co concentration correlated with worse clinical outcomes and high revision rate. Therefore, we advocate that patients with DePuy ASR XL implants be followed up more closely than those with other implants, especially with high acetabular abduction angle and serum Cr or Co concentration.

## Introduction

Total hip arthroplasty (THA) is currently one of the most commonly performed and successful surgeries in China, for which can restore function, alleviate pain, and greatly improve the quality of life of patients with hip problems. However, implant failure is common due to early wear and localized osteolysis [1]. Metal-on-metal (MoM) THA has emerged as an attractive alternative bearing surface, because of hard-on-hard bearing surfaces yielding a superior wear profile, preventing osteolysis, and improving implant longevity [2, 3]. Furthermore, MoM bearing allows for the implantation of femoral heads with larger diameters, thus increasing the head-to-neck ratio and jump height and theoretically improving the range of motion (ROM) while decreasing the risk of dislocation [4].

ASR XL is a MoM bearing surface implant that is widely used in China. The reasons for revision differ from those of other prostheses. The ASR acetabular component, although stable [5], is subject to more wear than other resurfacing devices and higher concentrations of cobalt (Co) and chromium (Cr) ions in patients’ blood and urine [6–8]. The incidence of pseudotumors after MoM arthroplasty is reported to be as high as 39 to 59% [9, 10].

The revision rate of this ASR XL articulation continues to rise in China. Concerns over the early performance of this design led our institution to stop using this device after less than 4 years. The present situation is in certain center which illuminates the usage of DePuy ASR articulation; however, their follow-up time is only 2 to 5 years. Moreover, as to this series report, in China, it has not yet referred.

From July 2006 to January 2010, we performed more than 100 surgeries using the ASR XL (DePuy, Warsaw, IN, USA) in our institution. We took a retrospective review of our mid-term clinical experiences with 74 consecutive DePuy ASR XL THA procedures performed by three senior orthopedic surgeons. Our study aimed to assess the implant design’s performance with respect to the following: (1) the demographic characteristics of patients who underwent MoM THA, (2) the relationship between blood metal ion levels and rate of early clinical failure, (3) the cumulative survival probability at the final follow-up, and (4) the risk factors of the revision surgery.

## Materials and methods

### Patients

We prospectively followed 98 patients (101 hips) after second-generation MoM for primary THAs from July 2006 to January 2010. Patient data are maintained in a database that includes observations from clinical examinations, radiological data, blood Co and Cr ion levels, and cross-sectional imaging. The data were analyzed retrospectively. Prior to the initiation of this study, approval was obtained from the local ethics committee and each patient provided informed consent.

In total, 25 hips were lost to follow-up; one patient was lost to high paraplegia (cervical spine fracture), and one died of causes unrelated to surgery; five patients received an ASR HR in one hip and an ASR THR in the other hip and were excluded from the study; and the rest 18 patients were out of contact during the follow-up. Overall, 74 prostheses were analyzed for Kaplan–Meier survivorship using clinical and radiographic outcome measures. Seventy-four patients (74 hips) had complete metal ion data for serial outcome measurements. This subset had concomitant clinical and radiographic parameters to substantiate our metal ion data.

### Surgery

All surgeries were performed by three senior surgeons. A posterolateral approach with detachment of the short external rotators was utilized in all procedures. The acetabulum was prepared by under-reaming by 1 mm. The ASR acetabular component is a CoCrMo alloy one-piece cup with proprietary porous coating. The outer surface of the cup consists of this porous coating with the addition of hydroxyapatite coating.

### Clinical evaluation

The Harris Hip Score (HHS) was determined at each follow-up visit by one physician Dr. Jisheng Ran. The ROM was calculated as part of the HHS. The Western Ontario and McMaster Universities (WOMAC) index were also determined based on a questionnaire.

### Biochemical evaluation

Venous whole blood samples were obtained to determine Co and Cr levels. Blood was initially sent to the Analysis and Testing Center of Zhejiang University. The Analysis and Testing Center utilized an octopole reaction system (ORS) inductively coupled plasma mass spectrometer (ICPMS) to measure the whole blood metal ion levels. The ORS-ICPMS method has been previously described by Pei [11].

### Radiological evaluation

Standardized anteroposterior (AP) and cross-table lateral radiographs of the pelvis and hip were assessed. All AP radiographs were taken with the legs in 15° internal rotation. The cup inclination angle in the frontal plane was measured against a horizontal reference line drawn along Kohler’s teardrop figures.

### Statistical analysis

Kaplan–Meier survivorship analysis was performed to determine the survival rates in the THA groups. Although the survival rates were not statistically compared, the differences between the revised and non-revised groups were compared. Normally distributed continuous variables (e.g., age, cup inclination, and femoral head diameter) were compared by independent *t* tests. Variables with skewed distributions (e.g., Co and Cr levels) were compared using the Wilcoxon–Mann–Whitney test. Valuable correlations were calculated using Cox regression and Omnibus tests of model coefficients after adjusting for age, gender, acetabular cup, inclination, femur head size, metal ion (Co and Cr) level, body mass index (BMI), and WOMAC index; these were used to make statistical comparisons of the revision rate between groups. The ROC curve was used to estimate the performance of cup inclination, Cr concentration, and Co concentration to revision surgery. The assumption of a valuable correlation was determined analytically for each model; if the interaction between the predictor and the log of the postoperative time was significant in the standard Cox regression model, a time-varying model was used. There were no missing data for the variables included in this study. All statistical analyses were performed using SPSS software.

## Results

Of the 74 patients, there were 36 (48.6%) males and 38 (51.4%) females, with a mean age of 56.12 ± 13.15 years. The primary diseases for the initial THA were 33 (44.6%) aseptic necrosis of femoral head (ANFH), 10 (13.5%) osteoarthritis (OA), 16 (21.6%) developmental dysplasia of the hip (DDH), 7 (9.5%) traumatic arthritis, and 8 (10.8%) other disease. The head size, cup size, and acetabular abduction angle of the prostheses were 45.12 ± 3.12 mm, 50.96 ± 3.90 mm, and 47.84 ± 6.35°, respectively. The average follow-up time was 88.46 months with the range of 23 to 114 months (Table 1).

**Table 1:** Patients’ characteristics

Characteristics	Patients (cases (%))
Gender	
Male	36 (48.6)
Female	38 (51.4)
Age, years, mean ± SD	56.12 ± 13.15
BMI (kg/m^2^), mean ± SD	23.37 ± 3.19
Primary disease	
ANFH	33 (44.6)
Osteoarthritis	10 (13.5)
DDH	16 (21.6)
Traumatic arthritis	7 (9.5)
Else	8 (10.8)
Revision surgery	
Yes	18 (24.3)
No	56 (75.7)
Head size (mm), mean ± SD	45.12 ± 3.12
Cup size (mm), mean ± SD	50.96 ± 3.90
Acetabular abduction angle (°), mean ± SD	47.84 ± 6.35
Serum Co concentration (μg/L), mean ± SD	11.90 ± 22.65
Serum Cr concentration (μg/L), mean ± SD	107.12 ± 30.16
Fellow-up time (month), mean [range]	88.46 [23–114]

*BMI* body mass index, *ANFH* aseptic necrosis of femoral head, *DDH* developmental dysplasia of the hip, *SD* standard deviation

**Fig. 1:**
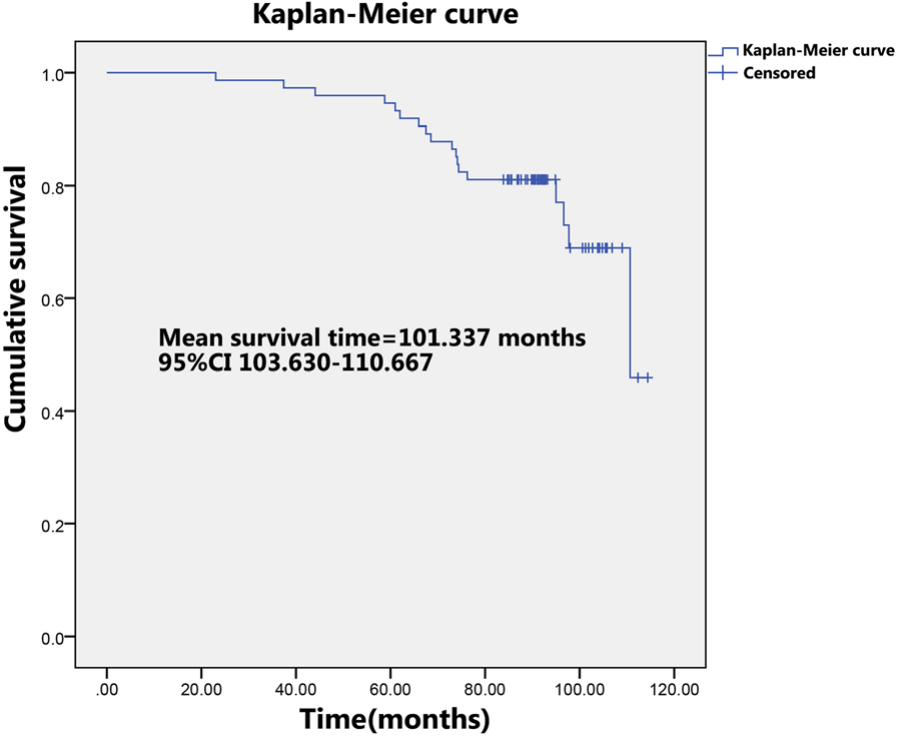
Kaplan–Meier curves for the ASR XL MoM prostheses after implantation

Within the follow-up time, there were 18 early failures (24.3%) of the ASR XL MoM THAs that required revision surgery (Table 1). The mean overall survival time of the prostheses was 101.34 months (95% CI 103.63–110.67 months). All the prostheses were functioning well at the first 2-year follow-up and the revision rate increased over the next 8 years (Fig. 1).

Patients who received revision surgery had worse joint function with significantly lower HHS and WOMAC index than non-revision patients (61.11 ± 6.68 vs 85.30 ± 9.16, *p* < 0.001; 61.00 ± 3.83 vs 79.04 ± 14.49, *p* < 0.001; respectively). The acetabular abduction angle of the revision group was significantly higher than that of the non-revision group (51.54 ± 3.30 vs 46.64 ± 6.65°, *p* = 0.004) while head size and cup size were comparable (45.61 ± 3.18 vs 44.96 ± 3.12 mm, *p* = 0.448; 51.56 ± 3.73 vs 50.77 ± 3.97 mm, *p* = 0.460; respectively). Further, we analyzed the serum Co and Cr concentration and found both Co and Cr concentrations were significantly higher in the revision group (21.04 ± 24.19 vs 8.96 ± 21.54 μg/L, *p* = 0.048; 124.48 ± 16.82 vs 104.18 ± 31.91 μg/L, *p* = 0.012; respectively), indicating a more severe metal abrasion (Table 2).

**Table 2:** Comparison between the revision patients and the non-revision patients

	Non-revision (*N* = 56)	Revision (*N* = 18)	*P* value
Sex (Male:female)	56 (26:30)	18 (10:8)	0.592
Age, years	56.61 ± 12.22	54.61 ± 16.01	0.579
BMI, kg/m^2^	22.98 ± 3.22	24.57 ± 2.84	0.066
Primary disease			
ANFH	25	8	0.365
Osteoarthritis	9	1	
DDH	13	3	
Traumatic arthritis	5	2	
Else	4	4	
Head size (mm)	44.96 ± 3.12	45.61 ± 3.18	0.448
Cup size (mm)	50.77 ± 3.97	51.56 ± 3.73	0.460
Acetabular abduction angle (°)	46.64 ± 6.65	51.54 ± 3.30	0.004
Serum Co concentration (μg/L)	8.96 ± 21.54	21.04 ± 24.19	0.048
Serum Cr concentration (μg/L)	104.18 ± 31.91	124.48 ± 16.82	0.012
Harris Hip Score	85.30 ± 9.16	61.11 ± 6.68	< 0.001
Western Ontario and McMaster Universities (WOMAC) index	79.04 ± 14.49	61.00 ± 3.83	< 0.001

*BMI* body mass index, *ANFH* aseptic necrosis of femoral head, *DDH* developmental dysplasia of the hip

**Fig. 2:**
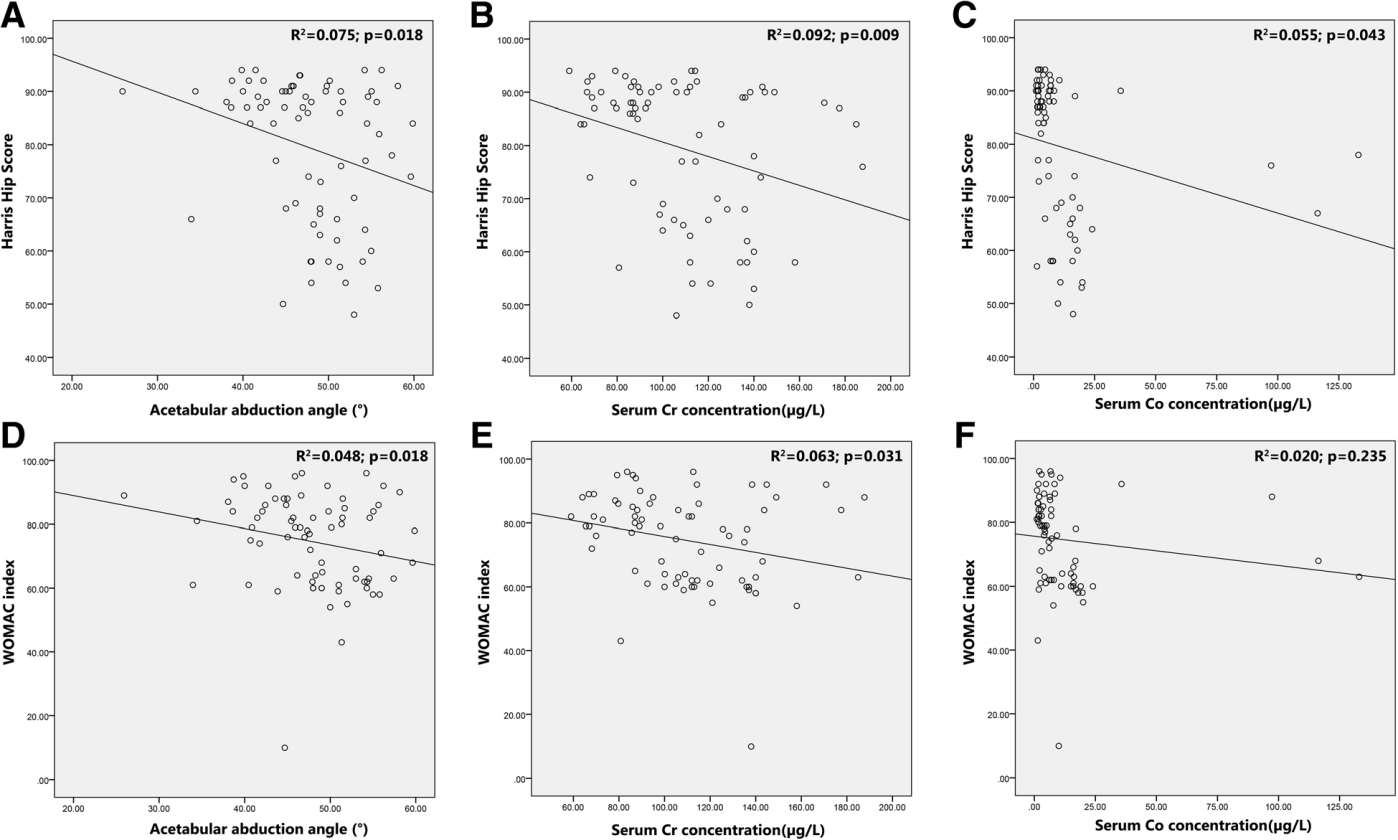
**a**–**f** Scatter diagram and correlation analysis of the acetabular abduction angle, serum Co and Cr concentration with HSS and WOMAC index. HSS: Harris Hip Score. WOMAC index: Western Ontario and McMaster Universities index

**Fig. 3:**
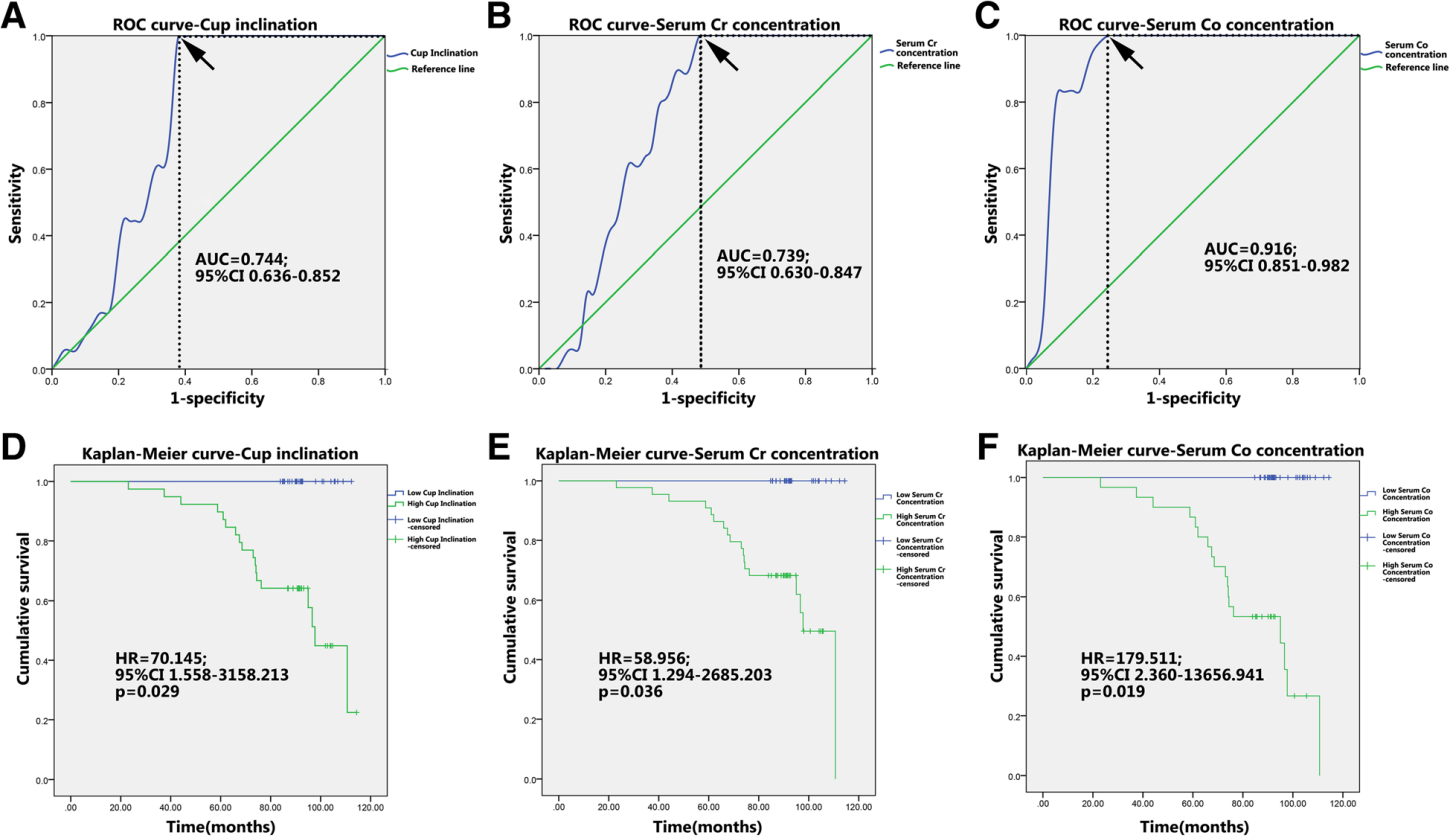
ROC curves and the Kaplan–Meier curves for the ASR XL MoM prostheses after implantation. **a**–**c** ROC curve analysis was applied to categorize the optimal cutoff values of acetabular abduction angle and serum Cr and Co concentration for revision surgery. Black arrow indicates optimal cutoff values. **d**–**f** Kaplan–Meier curves for the ASR XL MoM prostheses with different acetabular abduction angle and serum Cr and Co concentration. High acetabular abduction angle (> 47.80°), high serum Cr concentration(> 98.44 μg/L), and high serum Co concentration(> 6.95 μg/L)

Higher acetabular abduction angle and serum Cr concentration were significantly relevant to lower HSS (*R*^2^ = 0.075, *p* = 0.018; *R*^2^ = 0.092, *p* = 0.009; respectively) and WOMAC index (*R*^2^ = 0.048, *p* = 0.018; *R*^2^ = 0.063, *p* = 0.031; respectively) (Fig. 2a, b, d, and e). Higher serum Co concentration was also significantly relevant to lower HSS (*R*^2^ = 0.055, *p* = 0.043) but not to WOMAC index (*R*^2^ = 0.020, *p* = 0.235) (Fig. 2c, f). These data revealed that higher acetabular abduction angle and serum Cr and Co concentration may be risk factors of bad joint function, early prostheses failure, and subsequent revision surgery. To confirm this hypothesis, ROC curve analysis was applied to categorize the optimal cutoff values of acetabular abduction angle and serum Cr and Co concentration for revision surgery, which were settled as 47.80°, 98.44 μg/L, and 6.95 μg/L, respectively (Fig. 3a–c). Based on this setting, we classified the patients into groups of “high acetabular abduction angle (> 47.80°)”, “high serum Cr concentration (> 98.44 μg/L)” and “high serum Co concentration (> 6.95 μg/L)”. The Kaplan–Meier curves were then carried out to compare the overall survival (OS) of the prostheses between the higher and lower group in each parameter above. The results demonstrated that the OS with high acetabular abduction angle (HR = 70.145, 95% CI 1.558–3158.213, *p* = 0.029), high serum Cr concentration (HR = 58.956, 95% CI 1.294–2685.203, *p* = 0.036), and high serum Co concentration (HR = 179.511, 95% CI 2.360–13656.941, *p* = 0.019) decreased significantly than the lower groups (Fig. 3d–f).

**Fig. 4:**
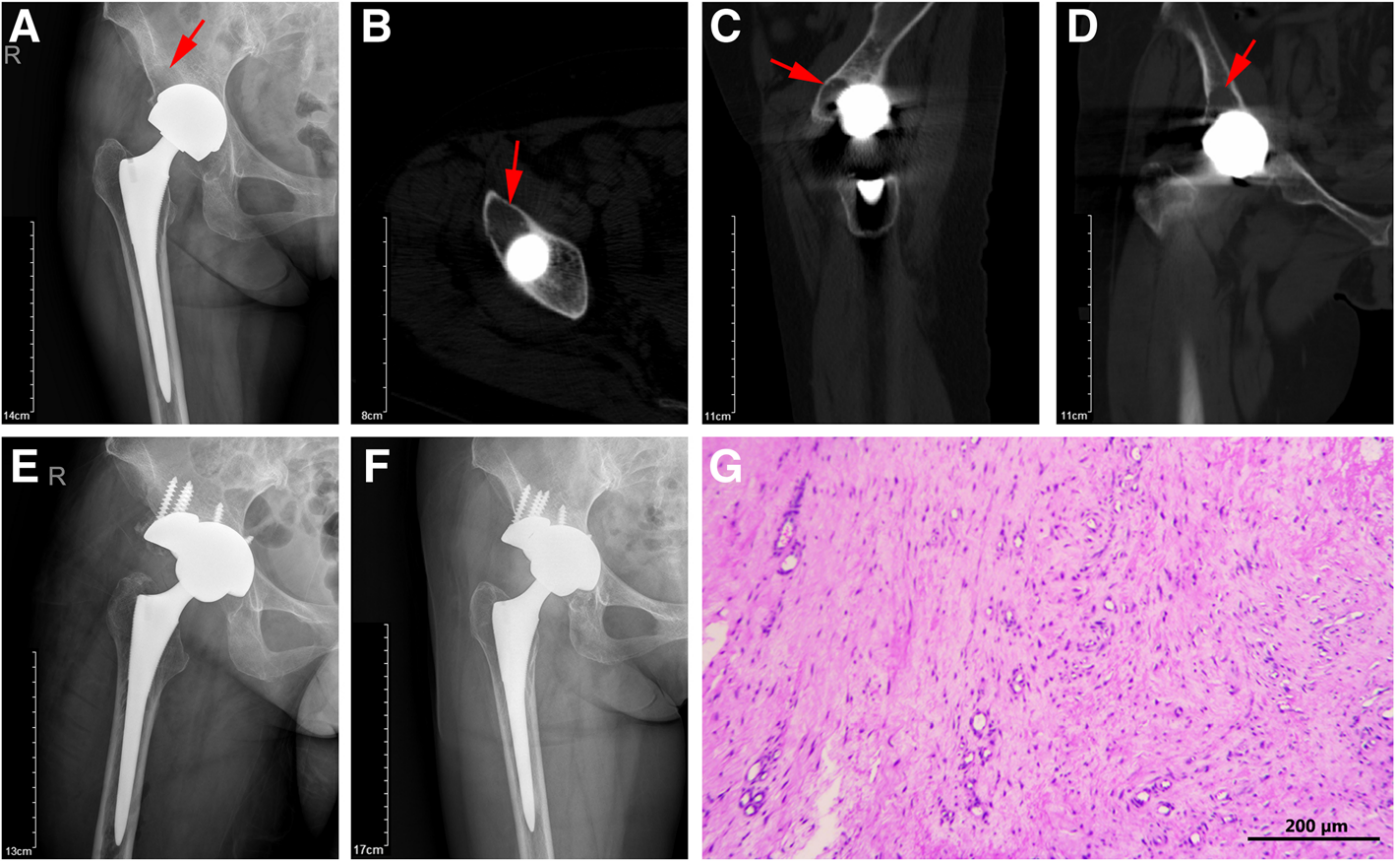
A typical revised case of the ASR XL MoM prostheses with cystic changes in periprosthetic bone. X-ray (**a**), cross section (**b**), sagittal section (**c**), and coronal section (**d**) of CT scan. Red arrow indicates bone cyst formation. X-ray images of 1 week (**e**) and 1 year (**f**) after revision surgery. HE staining of the periprosthetic soft tissue exhibited dense fibrous tissue with granuloma formation (**g**). Scale bar = 200 μm

In the revision procedures, cystic changes were noted in nearly all cases (Fig. 4a–d), 8 of 18 cases exhibited pseudotumor formation. Mild metal staining was noted in gross soft tissues. Aside from the two cases of gross loosening of the acetabular component, cups appeared mechanically stable; however, an osteolytic membrane was encountered around the outer rim in all cases (Fig. 4g). Less than 25% in-growth was noted on the explanted cups. Significant stress shielding of the retroacetabular bone was also noted. At 6 weeks after revision, all patients reported marked improvement in preoperative pain. At 1 year after revision, all patients were asymptomatic.

## Discussion

To investigate the relationships among acetabular cup inclination, head size, serum metal ion levels, and revision rate, we performed metal-on-metal THA in a consecutive series of patients. We also assessed self-reported hip function. Despite the theoretical advantages of increased head size and low-bearing surface wear, our senior surgeon handled THA with the DePuy ASR XL and had a 24.3% incidence of early revision.

Although MoM articulations possess numerous theoretical advantages in implant design, concerning revision rates have been reported in many MoM implant systems. The surgeon notification provided by DePuy in conjunction with the August 2010 recall of the device noted a 13% revision rate for the ASR XL MoM THA [12]. The revision rate, regardless of reason, reported in different studies of the ASR Hip System stratified by time from index surgery was 28.0% during the follow-up period of 6 to 7 years. Dastane et al. reported a 6-year failure rate of 25% for Articular Surface Replacement (ASR) resurfacing (DePuy, Leeds, UK) and one of 48.8% for ASR THA. The revision rate observed at our institute is close to the median value reported in their series, and our results are similar to previously reported studies that used the same acetabular construct.

The association between component orientation and bearing surface wear [13, 14], as well as blood metal ion concentration, is well documented. We observed an increased risk for elevated revision rate related to high cup acetabular abduction angle in the ASR THA group. Cup inclination was identified as a statistically significant factor in determining the revision rate in our patient cohort. Similarly, in three other studies, the suboptimal acetabular abduction angle was associated with a higher revision rate for ALTR or an increased early revision rate [15–17].

The issue of taper debris is associated with greater tissue damage than equivalent doses from the bearing surface [18], that is believed to be the main reason for higher metal ion concentrations in THAs [19], as the rates of bearing surface wear in MoM THAs and resurfacings are similar [20]. Corrosion is more often seen at the titanium (Ti)-CoCr interface; the Biomet ReCaps (Biomet Manufacturing, LLC in Warsaw) has a Ti-Ti-interface, which results in a lower Co release in Biomet MoM THAs using a Ti sleeve [21].

High wear rate is the major determinant of failure of large MoM hip implants, and edge loading has been identified as the most important predictor [22]. A large femoral head size in ASR XL THA is believed to be a risk factor for ALTR and an increased revision rate due to trunnionosis. In our study, both serum Co and Cr concentrations were not relevant to cup size (*p* = 0.205, *p* = 0.701, respectively) and head size (*p* = 0.667, *p* = 0.476, respectively). However, serum Cr concentration was proved to be correlated with the acetabular abduction angle (*p* = 0.014) in our study, in accordance with the findings of previous studies that found higher blood metal ion levels with decreased/increased abduction angles of the acetabular component [23, 24]. High acetabular abduction angle may lead to abnormal edge loading, resulting in high serum metal ion concentration.

Higher blood metal ion values clearly correlated with a higher revision rate, ALTR prevalence, and raised blood lymphocyte levels [25, 26]. With increasing concerns regarding the high failure rate of large-head MoM THAs, it is important to identify a safe upper limit for femoral head size in MoM THA. Concentrations of 5 μg/L and 7 μg/L have been used as thresholds for Co and Cr levels, respectively. We detected much higher concentrations of serum metal ions (Co and Cr), with the mean levels reaching 11.90 μg/L and 107.12 μg/L, respectively, especially in the revised group (Co and Cr levels were 21.04 μg/L and 124.48 μg/L, respectively). In our cohort, a 6.95 μg/L cutoff of Co level and a 98.44 μg/L cutoff of Cr levels were made based on ROC curve analysis, with 78.6% and 53.6% specificity, respectively, and both 100% sensitivity. The Kaplan–Meier curves further demonstrated that patients with high Co level (> 6.95 μg/L) or high Cr level (> 98.44 μg/L) exhibited significantly higher revision rate and shorter prostheses survival time. Our data re-emphasized the predictive value of serum Co and Cr level for early failure and revision surgery in patients with ASR XL MoM THAs.

Although we found a pseudotumor prevalence of 44.44% (8 in 18) in our study, we did not determine the potential correlation between the presence of a pseudotumor and other risk factors, including elevated metal ion levels and/or image demonstration after MoM THA. Hailer et al. [27] characterized the risk factors of pseudotumor formation; apart from Co level, which was discussed earlier, they found an inverse relationship between implant size and the formation of pseudotumors. Pseudotumors are a common complication of MoM arthroplasties, and elevated metal ion concentrations in the blood are also a common finding after such procedures [9, 28]. However, Bayley [29] indicated no association between the presence of pseudotumors and the potential risk factors analyzed herein, including elevated metal ion levels.

There are several limitations to our study. First, this was a retrospective, randomized study. The retrospective methodology limits data collection options; indeed, 26 hips were lost to follow-up during the study. Moreover, the retrospective used in this study design lends itself to several biases, including selection and recall. A second limitation is that, during the revision procedure, we observed the formation of a pseudotumor periprosthetic but did not collect any objective images, such as ultrasonography or the more sensitive metal artifact reduction sequence magnetic resonance imaging (MARS MRI). These could have clarified the prevalence of pseudotumors preoperatively and, thus, allowed us to better assess the formation of pseudotumors. A third limitation is the relatively small sample size. Between July 2006 and January 2010, our institution performed 3,000 THAs; however, only 200 hips received the ASR XL MoM THA (100 hips complied with the follow-up visits). In China, even more than one agency had done so many THAs, our follow-up data may not delegate for the integrity. Additionally, we did not evaluate the effect of the specific stem and cup combinations due to the large variety of stems used. Differences in the wear properties and metal ion release between taper designs have been described elsewhere [16]. Limiting the analysis to only bearing surface brands may oversimplify the results.

Thus, we advocate that patients with DePuy ASR XL implants be followed up more closely than patients with other implants, especially when symptomatic. Owing to the unusually high failure rate, poor early results with a relatively new implant design highlight the need for a national joint registry; indeed, an implant such as this one, with a poorer-than-expected survivorship, could have been more quickly identified and removed from the market. Further long-term, comparative studies of MoM and alternative bearing surfaces remain a priority for future research.

## Conclusions

Evaluation of the DePuy ASR XL articulation demonstrated increased rates of revision following a longer follow-up period. High acetabular abduction angle and serum Cr and Co concentration correlated with worse clinical outcomes and high revision rate. Therefore, we advocate that patients with DePuy ASR XL implants be followed up more closely than those with other implants, especially with high acetabular abduction angle and serum Cr or Co concentration.

## Abbreviations

ASR: Articular surface replacement
HHS: Harris Hip Score
MOM: Metal-on-metal
ROM: Range of motion
THA: Total hip arthroplasty
WOMAC: Western Ontario and McMaster Universities

## Authors’ information

Guojun Jin

Final degree: MD

Address: Department of Orthopedic Surgery, The Second Affiliated Hospital, School of Medicine, Zhejiang University, 88 Jie Fang Road, Hangzhou 310009, China.

PhD candidate, currently working in Taizhou First People’s Hospital.

E-mail: 19119025@sina.com

Jisheng Ran

Final degree: MD

Address: Department of Orthopedic Surgery, The Second Affiliated Hospital, School of Medicine, Zhejiang University, 88 Jie Fang Road, Hangzhou 310009, China.

E-mail: ranjisheng@zju.edu.cn

Weiping Chen

Final degree: MD

Address: Department of Orthopedic Surgery, The Second Affiliated Hospital, School of Medicine, Zhejiang University, 88 Jie Fang Road, Hangzhou 310009, China.

E-mail: cwpstks@126.com

Yan Xiong

Final degree: MD

Address: Department of Orthopedic Surgery, The Second Affiliated Hospital, School of Medicine, Zhejiang University, 88 Jie Fang Road, Hangzhou 310009, China.

E-mail: xiongyanbear@zju.edu.cn

Jiapeng Bao

Final degree: MD

Address: Department of Orthopedic Surgery, The Second Affiliated Hospital, School of Medicine, Zhejiang University, 88 Jie Fang Road, Hangzhou 310009, China.

E-mail: baojiapeng2007@aliyun.com
